# The nexus between internet use and consumption diversity of rural household

**DOI:** 10.1016/j.heliyon.2024.e35433

**Published:** 2024-07-30

**Authors:** Xue Gao, Junpeng Li

**Affiliations:** a School of Economics, Northeastern University at Qinhuangdao, Qinhuangdao, 066004, China; b School of Business Administration Northeastern University, Northeastern University, Shenyang, 110167, China; c Business School, Jiangsu Collaborative Innovation Center of Regional Modern Agriculture & Environmental Protection, Huaiyin Normal University, Huaiyin, 223300, China

**Keywords:** Internet use, The diversity of consumption, CFPS, Mechanism analysis

## Abstract

Exploring approaches to improve rural household well-being has always been an important task. This research investigates the effect of Internet use on rural household well-being measured by household consumption diversity, utilizing the 2016, 2018, and 2020 China Family Panel Studies survey data. To assess rural household consumption diversity, we employ the Simpson index and Shannon−Weaver index. To address the endogeneity of Internet use, we regress an instrumental variable-based two-stage least square (2SLS) method. The results show that Internet use substantially improves rural household consumption diversity. The disaggregated analysis suggests that low-income and small households in Central China benefit the most in consumption diversity improvement from using the Internet. Moreover, the mechanism analysis results show that household deposits and households’ beliefs about accessing commercial activities can positively mediate the relationship between Internet use and consumption diversity of rural household. Our findings provide new evidence for the literature on the role of Internet use in improving household consumption diversity in rural China.

## 1 Introduction

The diversity of household consumption expenditure helps improve household well-being, promotes the accumulation of family human capital, and fosters long-term rural development [1]. However, rural households in some developing countries face asymmetric information and limited access to product markets, thereby impeding the diversity of household consumption [2,3]. For instance, because of asymmetric information and poor product market access, households in rural Asia and Africa spend large proportions of their budgets to satisfy survival needs, such as food, water, shelter, and clothing [4,5]. The prioritization of survival needs over high-level needs (including education, travelling, and participating in recreational activities) has far-reaching implications for household well-being and overall flourishing in rural areas. Therefore, adopting feasible methods to diversify rural household consumption is essential.

Internet use, a key component of information and communications technology, could help reduce information asymmetry as it provides a wide range of information quickly and with low costs [6]. Rural households can benefit from the reduction in information asymmetry and gain more opportunities for nonfarm work [7], farmland transfer [8], higher agricultural sales [9], and better financial performance [10], thereby increasing their household income and deposits [11]. With the increase in household income and deposits, there may be an improvement in consumption diversity. In addition, Internet use could help improve product market access by easing online payments and shopping [12]. Specifically, household members can purchase goods and services through online payments (e.g. Paypal, Alipay, and WeChat Pay) and shopping platforms (e.g. Amazon, eBay, and Taobao), because online payments and shopping platforms enable them to overcome temporal and geographical limitations [13]. Therefore, Internet use has the potential to increase the diversity of rural household consumption.

Previous studies rarely analyse the impact of Internet use on rural household consumption diversity; despite the considerable research on the impacts of Internet use. In previous studies, the effects of Internet use on the subjective and objective well-being of individuals and households have received wide attention [14,15]. In terms of subjective well-being, Rahman et al. [14] discovered a positive relationship between Internet use and the subjective well-being of farmers. This positive relationship also applies to small-scale fishers in East Java, Indonesia [15].

In terms of objective well-being, scholars believed that objective well-being can be improved by using the Internet. For instance, based on the Chinese data, Zheng et al. [16] found that the utilization of the Internet has the potential to enhance agricultural income. Leng et al. [17] discovered a positive relationship between Internet use and household income diversification in China. Apart from income, the objective well-being of households can be measured by the diversity of household consumption; however, limited research explores the relationship between internet use and consumption diversification among rural households. One of the exceptions is the work of Vatsa et al. [18], who found that Internet use significantly promotes rural household consumption diversity. It is worth noting that the measurement of Internet use in their study solely focused on the Internet use of household heads; however, household Internet use depends on the decisions of all household members. To comprehensively evaluate the association between Internet use and household consumption diversity, it is necessary to consider the household-level Internet use. Thus, this study utilizes household-level Internet use and investigates its impact on the diversity of rural household consumption, along with exploring the underlying mechanisms.

This study focuses on China, which serves as an interesting case study. China has made great efforts to expand Internet coverage. Internet use penetration in the country has increased rapidly, from 37.70 % in 2011 to 73.09 % in 2021. Correspondingly, the number of Internet users exceeded 1 billion in 2021 [19]; however, Internet coverage in rural areas remains low compared to urban areas [18]. Furthermore, China aims to diversify rural residents’ consumption. According to national statistical data, for rural residents in China, the proportion of per capita basic expenditure (including food, tobacco, alcohol, clothing, and housing) in total expenditure decreased from 63.74 % in 2011 to 58.93 % in 2021, while the expenditure of higher-level needs (e.g. transportation and communication, education, and entertainment) in total expenditure increased from 30.14 % in 2011 to 35.44 % in 2021. However, compared with developed countries like the United States, the proportion that Chinese residents spend on higher-level needs in total consumption expenditure is still lower [20]. Thus, the impacts of Internet use on household consumption diversity in rural China are worth studying.

To examine the impact of household-level Internet use on household consumption diversity, we utilize the data from the China Family Panel Studies (CFPS) and apply an instrumental variable-based two-stage least square (2SLS) method to address the endogeneity associated with Internet use. The main empirical results show that Internet use substantially improves the rural household consumption diversity measured by the Simpson and Shannon−Weaver index. The disaggregated exploration suggests that low-income farmers in small households in Central China may benefit the most in consumption diversity improvement from using the Internet. In addition, the mechanism analysis results show that household deposits and households’ beliefs in accessing commercial activities positively affect the relationship between Internet use and household consumption diversity.

This paper makes three contributions to the literature. First, we attempt to investigate the impact and extent of Internet use on the diversity of household consumption in rural areas of China. Logically, Internet use can increase household consumption diversification by affecting household deposits and access to larger product markets. However, in practice, research on the association between them is scarce. Second, we focus on Internet use at the household level, not at the head of the household. Previous studies like Leng et al. [17], Vatsa et al. [18], and Zou et al. [8] only considered household head's Internet use, potentially leading to an underestimation of the impact of Internet use on household well-being. Different from previous studies, we measure household-level Internet use based on whether all members of a household, including the household head and other members, use the Internet. Third, we examine the mechanism between household-level Internet use and household consumption diversity, based on the CFPS data collected in the 2016, 2018, and 2020 waves.

The rest of this paper is organised as follows. Section 2 introduces the literature review, followed by the conceptual framework in Section 3. Section 4 presents the data, variables, and descriptive statistics, and Section 5 describes estimation strategies. Section 6 presents and discusses empirical results, while Section 7 draws conclusions and policy implications.

## 2 Literature review

### 2.1 Household consumption diversity and its determinants

Consumption diversity is often used to reflect economic welfare because diversified consumer categories are associated with better nutrition, cognition, emotional health, and innovation [21]. Consumption diversity is also essential for enterprise innovation and social development [22]. For instance, to meet consumers’ diverse and higher-level needs, some enterprises adopt advanced technologies and develop new products and services [22]. According to previous studies, there are various types of household consumption, including food consumption diversity [3], portfolio diversity [2], and total consumption expenditure diversity [23]. In contrast, food consumption diversity and portfolio diversity only focus on one aspect of household spending, failing to encompass other dimensions of household spending, while total consumption expenditure diversity is comprehensive. To measure various types of household consumption, scholars adopt two kinds of methods. The first method is to consider the increase in household consumption items [24], while the second method is to consider both the number of household consumption items and their respective shares in total household consumption expenditure [2,22,25]. For example, Johny et al. [25] exclusively relied on the number of consumed items. Zhou et al. [2] considered the number of consumption items and the consumption share of each item, utilizing the Simpson index and Shannon-Weaver index.

Scholars have further investigated factors influencing household consumption diversity. These factors generally include nonfarm employment [21], income diversity [22], crop production diversification [26], food market access [26,27], insurance [28] and financial investment [29]. Among them, nonfarm employment, income diversity, crop production diversification, and food market access positively affect household consumption diversity. Specifically, nonfarm employment increases rural household income, allowing rural residents to diversify their consumption [22]. Income diversity exerts a mixed impact on consumption diversity. Ma et al. [23] found that, for the lower quantiles (5th and 25th), income diversity has a significant positive effect on consumption expenditure diversity, while its positive effect is insignificant for the 75th quantiles. Islam et al. [26] concluded that improved access to markets effectively increases household consumption diversity.

In addition, factors like insurance and financial investment have uncertain effects on household consumption diversity. Insurance could reduce unpredicted risks confronted by people, increasing their consumption diversity [30]; however, some studies [28] identified an inverse correlation between social insurance and consumption diversity. In general, financial investment in stock assets positively affects individuals' expenditures because the rise of stock asset prices can increase residents’ property income [29]; however, Huang et al. [31] pointed out that the stock purchase behaviour could not improve household consumption diversity because the prices of stocks are unstable.

### 2.2 Impact of internet use on household consumption

The effect of Internet use on household consumption expenditure has received much attention. Relative studies can be divided into two branches. The first branch focuses on the impacts of Internet use on specific household expenditure items, including cosmetic expenditures [24], energy expenditures [7,32], risky financial assets investments [2], online shopping expenditures [33], transport expenditures [34,35], and food expenditure [10]. For instance, Zheng et al. [24] found that Internet use positively and significantly affects residents’ cosmetics expenditure in China, and a cosmetics expenditure gap exists between urban and rural residents. He et al. [7] found that Internet use has a significant and positive effect on the use of clean energy for cooking among Chinese farmers, and this impact is more substantial for farmers in the eastern region.

The second branch focuses on the impact of Internet use on overall household expenditure [13,36]. For instance, by estimating the 2018 China Household Finance Survey data, Zhang et al. [36] indicated that online payments positively impact overall household consumption in rural China, explaining that this positive impact could be attributed to the benefits of online payments in improving the convenience of consumption activities. Similarly, Zhao et al. [13] found that the mobile Internet penetration rate improves the overall consumption of rural households, and the impact is more substantial for socially vulnerable groups, such as the elderly, the less educated, and low-income people.

Furthermore, scholars have investigated the effect of Internet use on the diversity of household consumption because higher total household expenditure does not necessarily increase expenditure diversification [22]. For example, Zhou et al. [2] found that Internet use increases the diversity of risky financial asset portfolios, and the enhancement effect is more prominent for females than males. Vatsa et al. [18] focused on household heads' Internet use and analysed its effect on household expenditure diversity; however, only considering household heads’ Internet use would result in underestimating the impact of Internet use.

## 3 Conceptual frameworks

**Fig. 1:**
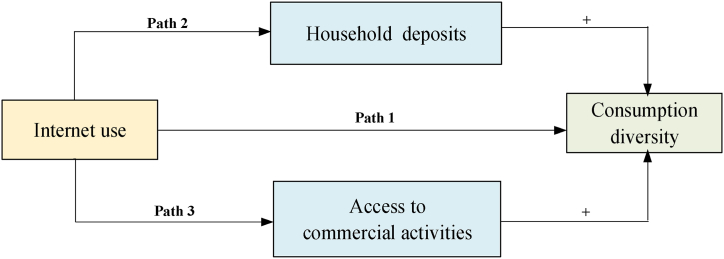
Impact pathways of Internet use on rural household consumption diversity.

Following the literature on Internet use [6,37] and household consumption diversity [18,22], we clarify the positive association between the two by delineating three possible channels, as shown in Fig. 1.

First, Internet use directly impacts rural household consumption diversity by facilitating the transmission of information on products and services. Relative to traditional tools (e.g. newspapers, radio, and television), Internet-based information transmission provides massive and timely information about goods and services. More importantly, according to each adopter's web browsing history, some Internet-based applications could judge individual preferences and provide personal product messages. Embraced by massive, timely, and accurate information, people are more likely to diversify their consumption.

Second, Internet use can increase rural household consumption diversity by increasing household deposits. The high level of deposits enables people to purchase a wider range of goods and services, thereby enhancing their consumption diversity [37]. Furthermore, numerous studies on the powerful information transfer function of the Internet have confirmed that Internet access improves household income in rural areas, including agricultural income [9,38], nonfarm-based salary income [39], entrepreneurial income [40], and property-based income [41]. The higher the household income, the greater the level of deposits. Therefore, Internet use can contribute to diversifying rural household consumption by increasing household deposits.

Third, online payments and shopping, specific manifestations of Internet use, can significantly reduce transaction costs, increasing people's consumption diversity. Online payments can save time because people do not have to spend time to go to the bank to withdraw money [13]. Online shopping could save the cost of money because it provides products and services at reduced costs compared to offline options. At the same time, the availability of lower prices online obviates the need to go out shopping, thereby enabling individuals to save on travel expenses. The reduction in both time and financial costs allows people to have more time and budget for consumption [42]; therefore, we can surmise that Internet use increases consumption diversity by saving the costs of time and money.

To summarise, Internet use can potentially elevate rural household consumption diversity. This paper uses the 2SLS model to analyse the CFPS data to empirically test this positive association.

## 4 Data, variables, and descriptive statistics

### 4.1 Data

The data used in this study is obtained from CFPS. The CFPS is a national and comprehensive social tracking survey project that aims to capture the changes in social, economic, demographic, and other relevant aspects in China. The CFPS survey targeted eligible households in 25 provinces of China and members of the sample households. As for the selection of eligible households, the CFPS survey employed a multistage sampling approach. In specific, the first stage was to select counties or districts in 25 provinces, the second stage was to select villages or communities, and the third stage was to select sample households. The CFPS data collection was launched in 2010, encompassing a sample of 16,000 households. Since the initial identification of sample households in 2010, CFPS has conducted five consecutive rounds of follow-up surveys on both household and individual samples in 2012, 2014, 2016, 2018, and 2020. Given the representativeness and richness of the CFPS data, a large number of scholars have employed it to investigate Chinese issues [23,42]. Following the extant literature, we use the CFPS data to carry out empirical analysis.

In this study, we use the CFPS data collected in the 2016, 2018, and 2020 waves. There are two reasons. The first reason is that the 2014, 2016, 2018, and 2020 waves provide more information on household consumption items than the 2010 and 2012 waves. For example, the 2010 and 2012 waves did not collect household expenditures for helping relatives, friends, and colleagues, which the 2014, 2016, and 2018 waves did. The second reason is that the 2014 wave did not collect information on how the Internet was accessed. Then, we take three steps to clean the CFPS data collected in the 2016, 2018, and 2020 waves. First, we kept 6553 rural household samples by removing the 7466 urban observations in the 2016 wave. In the 2018 and 2020 waves, we kept 6385 and 4512 rural household samples respectively after excluding urban observations. Second, we incorporated rural household samples from the 2016, 2018, and 2020 waves to obtain a total of 17,450 samples. Third, we removed 449 observations with missing values for key, mediating, and control variables. Thus, the final unbalanced panel data included 17,001 rural households.

### 4.2 Key variables

#### 4.2.1 Consumption diversity

The consumption diversity of rural households is evaluated using the Simpson and Shannon−Weaver indices. The two indices are widely used by scholars because they can consider both the items of household consumption and the share of each item in total household consumption expenditure [2,18,22,23]. There are similarities and differences between the two indices. The similarity is that the two indices are equal to 0 if a household only consumes one item. The higher the two indices, the more diverse the consumption. The difference is that the Simpson index gives greater weight to large consumption items compared to the Shannon−Weaver indices. Moreover, the Simpson index ranges from 0 to 1, while the Shannon−Weaver index ranges from 0 to lnm; where m refers to the number of household consumption items. In brief, the Simpson index is easier to calculate and interpret compared to the Shannon−Weaver index. Therefore, we mainly adopt the Simpson index to measure rural household consumption diversity and use the Shannon−Weaver index for the robustness test.

Following Zhou et al. [2], Vatsa et al. [18], and Ma et al. [23], the Simpson and Shannon−Weaver indices can be calculated as follows:

(1) $${Simpson}_{i}=1-\sum_{s=1}^{m}P_{i,s}^{2}$$

(2) $${Shannon-Weaver}_{i}=-\sum_{s=1}^{m}P_{i,s}\;\ln\;P_{i,s}$$

Where ${Simpson}_{i}$ and ${Shannon-Weaver}_{i}$ refer to the Simpson index and the Shannon−Weaver index of the household i, respectively. $P_{i,s}^{2}$ refers to the square of the share of the item s to total household consumption expenditure. $P_{i,s}$ refers to the share of the item s to total household consumption expenditure. $\ln\;P_{i,s}$ refers to the logarithm of the $P_{i,s}$. If the household i only consumes one item, the values of ${Simpson}_{i}$ and ${Shannon-Weaver}_{i}$ equal 0; as the number of expenditure items (m) increases, the values of ${Simpson}_{i}$ and ${Shannon-Weaver}_{i}$ are close to 1 and lnm, respectively. In this study, we categorized household consumption expenditure into 16 items, such as expenditures on food and clothes, tourism, education, and training (see Table A1 in Appendix).

#### 4.2.2 Internet use

Internet use is our key explanatory variable. Unlike previous literature focusing on household heads’ Internet use [18,24], we consider the Internet use at the household level. The household-level Internet use is measured as a binary variable, taking a value of 1 if all members of the household use the Internet and 0 otherwise.

### 4.3 Mediating variables

Aligning with our conceptual framework, we select two mediators; one refers to rural household deposits (M1), and the other represents rural households’ beliefs in accessing commercial activities via the Internet (e.g. using online payments and shopping online) (M2). M1 a continuous variable that represents the level of household wealth. M2 is a categorical variable (M2 = 1, 2, 3, 4, 5). If rural households believe that commercial activities like online payments and shopping online are unimportant in daily life, the value of M2 equals 1. If rural households believe that commercial activities are important, the value of M2 equals 5. The importance is constantly increasing from 1 to 5.

### 4.4 Control variables

We select control variables by drawing upon existing research on Internet use and household consumption [2,13,18,37]. Specifically, we use household heads' age, gender, education, household size, and health status to describe household demographic characteristics. We use household income, subsidies, car ownership, and financial product holdings to represent rural households' financial condition. Car ownership, a symbol of wealth, could increase household consumption [26]; thus, we control the impact of car ownership. Prior studies have also documented that favourable living conditions positively affect household energy consumption diversity [43]; thus, we select home cleanliness and clean cooking fuel access to represent rural households’ living conditions. Finally, we control for location indicators (e.g. Eastern, Central, and Western China).

### 4.5 Descriptive statistics

Table 1 shows the definitions and descriptive statistics of the variables used in our empirical analysis. The average value of the Simpson index in our sample is 0.52. The mean of the Shannon–Weaver index is 1.23. These results indicate that rural household consumption diversity needs to be improved. As for household-level Internet use, the proportion of households in which every household member uses the Internet is 36 %. This result is similar to the macro statistics stemming from the ‘43rd China Statistical Report on Internet Development’ [44], which indicates that Internet use penetration in rural areas is 38.4 %. This result also suggests that Internet adoption in China's rural areas requires increased popularisation. Table 1 also shows that the average age of rural household heads is 51 years, indicating that aged farmers dominate rural households in our sample. Furthermore, most of the sampled household heads are male, and the average education level is primary school, suggesting that most rural household heads tend to be poorly educated. The average household size is 4, and 20 % experience illness shocks. The average household income is 17.4 thousand CNY per capita. On average, 74 % and 39 % of rural households receive government subsidies and own a car, respectively. Only 1 % of households own financial products. The average level of home cleanliness is 3.43, and approximately 54 % of households use clean fuels.

**Table 1:** Variable definitions and descriptive statistics.

Variables	Definitions	Mean	S.D.
*Dependent variables*			
Simpson index	Household consumption diversity measured by the Simpson index	0.52	0.22
Shannon-Weaver index	Household consumption diversity measured by the Shannon-Weaver index	1.23	0.46
*Key variable*			
Internet use	1 if a rural household uses the Internet, 0 otherwise	0.36	0.48
*Control variables*			
Age	Age of household head (HH) (years)	51.23	14.09
Gender	1 if HHis male, 0 otherwise	0.57	0.49
Education	The education level of HH (1 = illiteracy, 2 = primary school, 3 = junior high school, 4 = senior high school, 5 = undergraduate college and above)	2.03	0.40
Household size	Residential population count within the household	3.97	2.01
Health status	1 if at least one family member falls ill, 0 otherwise	0.20	0.40
Household income	Total annual household income (10,000 yuan/capita)^a^	1.74	4.66
Car ownership	1 if household owns a car, 0 otherwise	0.39	0.48
Subsidies	1 if household receives government subsidies (e.g., subsistence allowance), 0 otherwise	0.74	0.43
Financial product holdings	1 if household holds financial products, 0 otherwise	0.01	0.09
Home cleanliness	Home cleanliness level: 1 = very untidy to 7 = very tidy	3.43	2.05
Clean cooking fuel access	1 if household uses clean fuels (e.g., electricity, natural gas, and solar energy) for cooking; 0 otherwise	0.54	0.49
Eastern region	1 if the household is situated in eastern China, 0 otherwise	0.38	0.48
Central region	1 if the household is situated in central China, 0 otherwise	0.25	0.43
Western region	1 if the household is situated in western China, 0 otherwise	0.36	0.48
IV	1 if the household believes that the Internet is a vital source of information, 0 otherwise	0.47	0.49
*Mediating variables*			
M1	Household deposits (10,000 yuan)^a^	1.93	7.15
M2	Households' beliefs in accessing commercial activities via the Internet: 1 = very unimportant to 5 = very important	2.41	1.61
Sample size		17,001	

Note: S.D. refers to standard deviation.

a Yuan is a Chinese currency.

## 5 Estimation strategies

### 5.1 Two-stage least square (2SLS) model

Internet use at the household level tends to be influenced by observed (e.g. household size, car ownership, and household income) and unobserved (e.g. household members’ innate abilities and enthusiasm regarding Internet use) factors [17,18]; therefore, Internet use is associated with observed and unobserved endogeneities [11]. To solve the endogeneity inherent in Internet use, scholars have employed the propensity score matching model [37], the endogenous treatment regression (ETR) model [45], and the endogenous ordered probit model [17]; however, these models apply to cross-sectional data. Considering the panel data used in this paper and the endogeneity inherent in Internet use, we follow Sun et al. [46] and Giovanis [47] and estimate the 2SLS method to determine the association between Internet use and household consumption diversity in rural China. Following Wooldridge [48], our 2SLS model contains two stages. The first stage can be specified as follows:

(3) $${Iu}_{it}=\alpha_{1}{IV}_{it}+\alpha_{2}X_{it}+\mu_{i}+\varepsilon_{it}$$

where the subscript i=1,2……N indicates the ith rural household; the subscript t= 1, 2, and 3 index the years 2016, 2018, and 2020, respectively. ${Iu}_{it}$ is the key independent variable, Internet use. ${IV}_{it}$ denotes the instrumental variable, and $X_{it}$ is a vector of control variables. $\mu_{i}$ is the unobserved random variable representing the intercept term of the individual heterogeneity. $\varepsilon_{it}$ is the disturbance term, while $\alpha_{1}$ and $\alpha_{2}$ are parameters to be estimated. According to model (3), we can obtain the predicted value $\hat{I}u_{it}$.

Following Vatsa et al. [18], we use households’ beliefs in the importance of acquiring information via the Internet as an IV. The logic is that if a household believes that the Internet is critical for obtaining the necessary information, they are more likely to use it; thus, the selected IV could be associated with household-level Internet use. To confirm the statistical validity of the IV, we adopt the weak instrumental variable test. Table A2 in Appendix presents the weak instrumental variable test results. The bottom of Table A2 shows that the critical values of the Cragg–Donald Wald *F*-statistic significantly exceed the critical values at the 10 % significance level, indicating no weak instrumental variables.

The second stage can be specified as follows:

(4) $${Simpson}_{it}=\beta^{*}\hat{I}u_{it}+\beta_{2}X_{it}+\mu_{i}+\varepsilon_{it}$$

Where ${Simpson}_{it}$ denotes the Simpson index. $\beta^{*}$ and $\beta_{2}$ are parameters to be estimated. $\hat{I}u_{it}$ are the predicted values from Equation (3), and the meanings of other variables are the same as those in Equation (3). By regressing Equations (3), (4), we can solve the endogenous bias and obtain a consistent estimate of $\beta^{*}$.

### 5.2 Mediating effect model

This study also investigates the channels through which Internet use affects household consumption diversity, which calls for mediating analysis. Following previous studies [49,50], we apply the mediating effect model to conduct mediating analysis, which contains three steps. The first step can be specified as follows:

(5) $${Simpson}_{it}=\alpha_{0}+\alpha_{1}{Iu}_{it}+\alpha_{2}X_{it}+\varepsilon_{it}$$

Where ${Simpson}_{it}$, ${Iu}_{it}$, and $X_{it}$ are the same as defined above. $\alpha_{0}$ is an intercept term, $\alpha_{1}$, $\alpha_{2}$ are coefficients, and $\varepsilon_{it}$ is the random residual term. If $\alpha_{1}$ is statistically significant, Internet use substantially affects rural household consumption. We then test the second and third steps, specified as follows:

(6) $${Me}_{it}=\beta_{0}+\beta_{1}{Iu}_{it}+\beta_{2}X_{it}+\varepsilon_{it}$$

(7) $${Simpson}_{it}=\gamma_{0}+\gamma_{1}{Iu}_{it}+\gamma_{2}{Me}_{it}+\gamma_{3}X_{it}+\varepsilon_{it}$$

Where ${Me}_{it}$ are the mediating variables, including M1 and M2. $\beta_{0}$ and $\gamma_{0}$ are intercept terms. $\beta_{1}$, $\beta_{2}$, $\gamma_{1}$, $\gamma_{2}$, and $\gamma_{3}$ are coefficients, and $\varepsilon_{it}$ is the random residual term. A significant $\beta_{1}$ in Equation (6) and $\gamma_{2}$ in Equation (7) indicate the existence of a mediating effect. In Equation (7), a significant $\gamma_{1}$ suggests the existence of the partial mediating effect, meaning that Internet use has direct and mediating effects on household consumption diversity. Otherwise, a complete mediating effect exists, meaning that Internet use only mediates household consumption diversity.

## 6 Results

### 6.1 Baseline analysis

#### 6.1.1 The first stage of 2SLS

Table 2 presents the estimates of the first stage of 2SLS in the second column, where the coefficient of age is positive and significant. This result indicates that households headed by older farmers are more likely to use the Internet. In modern rural China, elderly parents tend to live alone without their children around them [51]. To improve the convenience and visualisation of mutual communication, young family members tend to provide their parents with Internet access. The coefficient of education is positive and significant, showing that improving the household head's education can increase the likelihood of using the Internet. This result is consistent with Ma and Wang [27] and Zhu et al. [37]. Zhu et al. [37] indicated that the promotion of education enables farmers to recognise the Internet's importance in improving their economic performance and life quality, thus encouraging them to use it. Household income and financial product holdings are positively associated with Internet use, indicating that wealthy households are more likely to use the Internet. This result coincides with Cheremoshkina [52] and Christelis et al. [53], who found out that financial market access and purchasing financial products positively affects the use of the Internet. In addition, the IV's impact on Internet use is positive and significant at the 1 % level, indicating that family members are more likely to use the Internet if they believe it is an essential source of information acquisition.

**Table 2:** Impact of household-level Internet use on consumption diversification proxied by the Simpson index: 2SLS method estimates.

Variables	Internet use	Simpson index
Internet use		0.083 (0.026)^c^
Age	0.016 (0.004)^c^	−0.007 (0.002)^c^
Gender	−0.141 (0.182)	−0.025 (0.114)
Education	0.039 (0.009)^c^	0.0002 (0.006)
Household size	−0.003 (0.004)	0.008 (0.002)^c^
Health status	−0.002 (0.012)	−0.009 (0.008)
Household income (ln)	0.019 (0.006)^c^	0.010 (0.004)^c^
Subsidies	−0.007 (0.010)	0.006 (0.007)
Car ownership	0.016 (0.011)	0.016 (0.007)^b^
Financial product holdings	0.087 (0.049)^a^	0.042 (0.031)
Home cleanliness	−0.0003 (0.002)	0.002 (0.001)^a^
Clean cooking fuel access	−0.004 (0.011)	0.012 (0.007)^a^
Eastern region	−0.181 (0.098)	−0.088 (0.062)
Western region	0.086 (0.137)	−0.025 (0.086)
IV	0.231 (0.010)^c^	
Constant	−0.529 (0.223)^c^	0.842 (0.142)^c^
$R^{2}$	0.157	0.122
Observations	17,001	17,001

Note: Robust standard errors are presented in parentheses. The reference region is Central China.

a < 0.10.

b < 0.05, and.

c < 0.01.

#### 6.1.2 The second stage of 2SLS

Column 3 of Table 2 presents the results of the second stage. Internet use, the key explanatory variable, significantly positively affects the Simpson index, indicating that Internet use can improve rural household consumption diversity. This result is similar to the findings of Vatsa et al. [18], who focused on the effect of household heads' Internet use on household consumption diversity. Although both studies demonstrate positive coefficients for Internet use, there exists a disparity in the magnitudes of these coefficients. In particular, the Internet use coefficient in our study is 0.083, indicating that Internet use can help increase the Simpson index by 0.083 points. Regarding the average Simpson index of households in which not all members use the Internet (i.e. 0.482), the impact of Internet use on the Simpson index translates to an increase of 17.2 %. By contrast, the corresponding rate Vatsa et al. [18] reported is only 6.4 %. The dramatic differences in the effect magnitude of Internet use show that its impact on rural household consumption diversity will be underestimated if only household heads’ Internet use is considered.

The coefficient of household income is significantly positive, suggesting that higher income brings diversified consumption; this finding is consistent with Ma et al. [23], who determined that income increase is associated with higher consumption diversity. The coefficient of car ownership is significantly positive at 0.016, meaning that car ownership increases the Simpson index by 0.016 points. Regarding the average Simpson index among carless households (i.e. 0.492), the increase of 0.016 points is equivalent to a 3.2 % increase. Owning a car is beneficial for rural households to travel and shop in remote product markets, allowing farmers to diversify their consumption. The coefficient of home cleanliness is positive and significant, meaning that the consumption diversity is higher in households characterized by cleanliness. Using clean cooking fuel also has a significantly positive effect on the Simpson index. The probable reason is that using clean cooking fuel can improve health and living conditions [54], which helps farmers save money from medical expenditures and diversify their consumption.

### 6.2 Robustness analysis

To reinforce the reliability of our main empirical results, we use the Shannon–Weaver index as an alternative measure of household consumption diversity and assess its association with Internet use using the 2SLS model. Table 3 presents the results, showing that Internet use exerts a positive and statistically significant impact on the Shannon–Weaver index. This result suggests that the positive correlation between household consumption diversity and Internet use remains consistent. The coefficient of Internet use is 0.106, meaning that Internet use increases the Shannon–Weaver index by 0.106 points. Given the average Shannon–Weaver index of 1.123, the impact of Internet use on the Shannon–Weaver index translates to an increase of 9.4 %. For comparison, the impact of Internet use on the Simpson index is comparatively higher than its impact on the Shannon-Weaver index.

**Table 3:** Impact of household-level Internet use on consumption diversity proxied by the Shannon-Weaver index: 2SLS method estimates.

Variables	Internet use	Shannon-Weaver index
Internet use		0.106 (0.047)^b^
Age	0.016 (0.004)^c^	0.051 (0.004)^c^
Gender	−0.141 (0.182)	−0.034 (0.209)
Education	0.039 (0.009)^c^	−0.113 (0.010)
Household size	−0.002 (0.004)	0.008 (0.004)^a^
Health status	−0.001 (0.012)	−0.001 (0.014)
Household income (ln)	0.019 (0.006)^a^	0.009 (0.007)
Subsidies	−0.007 (0.010)	0.038 (0.012)^c^
Car ownership	0.016 (0.011)	0.385 (0.013)^c^
Financial product holdings	0.087 (0.049)^a^	0.150 (0.057)^c^
Home cleanliness	−0.0003 (0.002)	−0.0003 (0.002)
Clean cooking fuel access	−0.004 (0.011)	0.028 (0.013)^b^
Eastern region	−0.171 (0.098)	−0.113 (0.113)
Western region	0.085 (0.137)	−0.056 (0.157)
IV	0.231 (0.009)^c^	
Constant	−0.529 (0.223)^b^	−1.323 (0.259)^c^
$R^{2}$	0.158	0.386
Observations	17,001	17,001

Note: Robust standard errors are presented in parentheses. The reference region is Central China.

a < 0.10.

b < 0.05, and.

c < 0.01.

### 6.3 Heterogeneity analysis

To deepen our understanding, we conduct heterogeneity analysis by region, household size, and household income levels. Table 4 shows the estimates disaggregated by region, indicating that the coefficients of Internet use for Central and Western China are significant and positive, while the coefficient is insignificant for Eastern China. By contrast, the coefficient of Internet use for Central China is bigger, meaning that rural households in Central China benefit the most in consumption diversity improvement from using the Internet.

**Table 4:** Heterogeneity analysis: region.

Variables	Eastern	Central	Western
Internet use	0.069 (0.045)	0.104 (0.051)^a^	0.083 (0.041)^a^
Controls	Yes	Yes	Yes
Constant	0.730 (0.239)^a^	0.645 (0.265)^a^	0.705 (0.223)^a^
$R^{2}$	0.156	0.166	0.167
Observations	6588	4321	6092

Note: Robust standard errors are presented in parentheses.

a < 0.05.

Table 5 shows the estimates of the impact of Internet use on household consumption diversity by household size. In the second column of Table 5, the coefficient of Internet use is positive and significant, indicating that Internet use significantly affects household consumption diversity among small households. Conversely, the insignificant coefficients of Internet use in the third and fourth suggest that Internet use cannot improve the consumption diversity of medium and large households.

**Table 5:** Heterogeneity analysis: household size.

Variables	Small^a^	Middle^b^	Large^c^
Internet use	0.099 (0.035)***	0.069 (0.047)	0.125 (0.100)
Controls	Yes	Yes	Yes
Constant	0.796 (0.177)***	0.839 (0.262)***	0.793 (0.469)*
$R^{2}$	0.151	0.143	0.143
Observations	10,782	4674	1545

Note: Robust standard errors are presented in parentheses. ***< 0.01 and *< 0.10.

a “Small” represents the household with a population not bigger than 4.

b “Middle” represents the household with a population between 4 and 6.

c “Large” represents the household with a population of bigger than 6.

Table 6 shows the results of the household income heterogeneity of Internet use's impact. Specifically, the coefficient of Internet use is significantly positive for low- and high-income rural households; however, it is not significant for middle-income rural households. The probable reason is that the spending power and concept for low-income rural households are improved by using the Internet, encouraging them to diversify their consumption expenditure. High-income rural households using the Internet can gain more information on products and services and exposure to new things; thus, they can diversify their consumption. From the perspective of effect size, the positive impact on low-income rural households surpasses that observed in other groups. In other words, the consumption diversity of low-income rural households will improve significantly via Internet use compared to medium and high-income households. This finding is consistent with Ma et al. [23], indicating that the Internet helps reduce the consumption diversity gap among different income groups.

**Table 6:** Heterogeneity analysis: household income levels.

Variables	Low^a^	Medium^b^	High^c^
Internet use	0.291 (0.099)***	0.009 (0.039)	0.105 (0.050)**
Controls	Yes	Yes	Yes
Constant	1.055 (0.376)***	0.689 (0.265)***	1.234 (0.341)***
$R^{2}$	0.177	0.106	0.130
Observations	5659	6611	4731

Note: Robust standard errors are presented in parentheses. ***< 0.01 and **< 0.05.

a “Low” represents the household income less than the 25th income percentiles.

b “Medium” represents the household income between the 25th and 75th income percentiles.

c “High” represents the household income not less than 75th income percentiles.

### 6.4 Mechanism analysis

Table 7 presents the estimation results from the mediating effect models, where the second, third, and fourth columns display the mediating variable of rural household deposits (M1). The results in the fifth, sixth, and seventh columns are for the mediating variable of rural households’ beliefs in accessing commercial activities (M2).

**Table 7:** Estimation results for the mediation effect model.

Variables	M1 mediating			M2 mediating		
	Simpson index	M1	Simpson index	Simpson index	M2	Simpson index
Internet use	0.037 (0.004)^a^	0.200 (0.086)∗∗	0.036 (0.004)^a^	0.036 (0.004)^a^	1.492 (0.024)^a^	0.027 (0.005)^a^
M1			0.006 (0.000)^a^			
M2						0.006 (0.001)^a^
Controls	Yes	Yes	Yes	Yes	Yes	Yes
Constant	0.464 (0.011)^a^	5.137 (0.232)^a^	0.435 (0.011)^a^	0.459 (0.011)^a^	2.395 (0.066)^a^	0.444 (0.012)^a^
$R^{2}$	0.157	0.234	0.163	0.157	0.461	0.158
Observations	17,001	17,001	17,001	17,001	17,001	17,001
Sobel test		0.001 (0.000)^b^		0.009 (0.002)^a^		
Aroian test		0.001 (0.000)^b^		0.009 (0.002)^a^		
Goodman test		0.001 (0.000)^b^		0.009 (0.002)^a^		

Note: Robust standard errors are presented in parentheses.

a < 0.01 and.

b < 0.05. M1 refers to the logarithm of household deposits, and M2 refers to households' beliefs in accessing commercial activities via the Internet.

The results in Table 7 suggest that M1 and M2 significantly mediate between Internet use and the Simpson index. Specifically, the coefficient of Internet use in the third column indicates that Internet use positively affects M1, while the coefficient of M1 in the fourth column shows that M1 positively affects the Simpson index. As shown in the sixth and seventh columns, Internet use also positively affects M2, which positively affects the Simpson index; thus, M1 and M2 positively mediate the relationship between Internet use and household consumption diversity. Furthermore, the Sobel test, Aroian test, and Goodman test located in the lower part of Table 7 are all significant, sufficiently proving the existence of mediating effects of M1 and M2.

The mediation analysis suggests that M1 and M2 positively mediate the nexus between Internet use and household consumption diversity. As mentioned in this study's conceptual frameworks, improved economic conditions achieved via Internet use could allow people to purchase more goods and services, thus diversifying their consumption. Finally, the reduction in time and material costs caused by online payment and online shopping encourages people to consume more items.

## 7 Conclusion and policy implications

Household consumption diversity is critical for improving rural residents' life quality and rural development; however, consumption diversity for rural households is constrained by poor information access. Therefore, improving rural households’ information access is critical. Internet use may be an effective strategy to enhance information accessibility and increase household consumption diversity in rural areas; however, the extant literature has largely overlooked the nexus between Internet use and rural household consumption diversity.

Focusing on household-level Internet use rather than household heads' Internet use, this study explores the relationship between Internet use and rural household consumption diversity by utilizing 2016, 2018, and 2020 CFPS data. Based on the panel data, we use the 2SLS model to solve the endogeneity inherent in Internet use. This study measures rural household consumption diversity mainly through the Simpson index. The empirical results show that Internet use has a significantly positive impact on the Simpson index; specifically, the consumption diversity of rural households with all members using the Internet exhibits a 17.2 % higher level than other households. This result is dramatically larger than the 6.4 % derived by Vatsa et al. [18], who only focused on the effect of rural household heads' Internet use on household consumption diversity. Thus, we can infer that the impact of Internet use on rural household consumption diversity will be underestimated if we only consider rural household heads’ Internet use. The effect of Internet use remains robust when assessing the diversity of household consumption through the application of the Shannon-Weaver index. Furthermore, control variables, including household income, car ownership, home cleanliness, and clean cooking fuel access, positively affect rural household consumption diversity.

The disaggregated analysis suggests that low-income and small households in Central China benefit the most in consumption diversity improvement from using the Internet. Regarding the impact mechanism, the results confirm that rural household deposits and their beliefs in accessing commercial activities mediate between Internet use and household consumption diversity.

Our findings can provide significant policy implications for China aiming to increase rural household well-being. The policy proposals have four aspects. First, the positive impact of Internet use on rural household consumption diversity reminds policy designers to strengthen investments in constructing and improving rural telecommunication infrastructures. Second, based on the improvement of rural telecommunication infrastructures, it is necessary to improve rural residents' enthusiasm and ability to effectively use the Internet. Thus, governments should make rural residents feel the practicality and convenience of Internet use through formal and informal education, such as organising courses, providing volunteer services, and distributing learning materials. Third, to improve the usage of Internet, the governments can provide guidance to Internet companies in developing and promoting applications tailored for rural residents. Fourth, China's central government should pay attention to the regional differences in promoting Internet use in rural areas. Reinforcing the promotion of Internet use in Central and Western China is meaningful.

It should be noted that this study has two limitations. First, due to data limitations, we cannot investigate the effects of Internet use for different purposes, such as learning, working, and entertainment, on rural household consumption diversity. In practice, rural households could use the Internet to do many things, such as chatting with friends and relatives, browsing newspaper sites, and shopping online; such types of Internet use may exert different effects on rural household consumption. Second, apart from household deposits and households’ beliefs about accessing commercial activities, we are unable to investigate any other mediating effects like online lending. Future research may bridge these limitations when data become available.

## Data availability statement

The authors do not have permission to share data.

## Funding statement

This study was supported by the 10.13039/501100003787Hebei Natural Science Foundation (G2022501005), the 10.13039/501100012226Fundamental Research Funds for the Central Universities (N2323004).

## Ethics approval and consent to participate

Not applicable.

## CRediT authorship contribution statement

**Xue Gao:** Writing – original draft, Software. **Junpeng Li:** Conceptualization.

## Declaration of competing interest

The authors declare the following financial interests/personal relationships which may be considered as potential competing interests: Xue Gao reports financial support was provided by the 10.13039/501100003787Hebei Natural Science Foundation. Xue Gao reports financial support was provided by the Fundamental Research Funds for the Central Universities. If there are other authors, they declare that they have no known competing financial interests or personal relationships that could have appeared to influence the work reported in this paper.

## Figures and Tables

**Table A1:** Definitions and descriptive statistics of consumption expenditure items

Variables	Definition	Mean	S.D.
Expenditure on food and clothes	Expenditure on food outside and clothes (100 yuan) ^a^	39.88	65.22
Expenditure on entertainment	Expenditure on entertainment (e.g., newspapers, magazines, and movies) (100 yuan)	1.27	5.05
Expenditure on tourism	Expenditure on entertainment (e.g., transportation, accommodation, and scenic spot) (100 yuan)	3.52	19.13
Expenditure on heating	Expenditure on heating services (100 yuan)	1.27	5.05
Expenditure on housing	Expenditure on housing (e.g., decoration, maintenance, and expansion) (100 yuan)	48.54	313.35
Expenditure on car	Expenses on car (e.g., purchase, maintenance, and repair) (100 yuan)	32.11	238.27
Expenditure on transportation and communication	Expenditure on transportation (e.g., bicycles and electric bicycles) (100 yuan)	11.47	25.02
Expenditure on durable goods	Expenses on durable goods (e.g., computers, home appliances, and jewelry) (100 yuan)	14.20	90.83
Expenditure on education and training	Expenditure on education and training (100 yuan)	36.29	75.89
Expenditure on medical care	Expenditure on medical care (100 yuan)	52.93	156.45
Expenditure on body-building	Expenditure on body-building (e.g., equipment, course, and health care products) (100 yuan)	1.56	12.35
Expenditure on beauty	Expenditure on beauty (e.g., cosmetics, haircut, and massage) (100 yuan)	4.19	10.47
Expenditure on insurance	Expenditure on insurance (e.g., automobile insurance, property insurance, and life insurance) (100 yuan)	11.44	42.08
Expenditure for helping relatives	Expenditure for providing financial support to relatives (100 yuan)	10.03	60.12
Expenditure for helping friends and colleagues	Expenditure for providing financial support to friends and colleagues (100 yuan)	1.90	16.68
Expenditure on others	Expenditure on others (e.g., housekeeping services, lotteries, and fines) (100 yuan)	1.61	9.14
Sample Size		17,001	

^a^ Yuan is a Chinese currency.

**Table A2:** Regression results in the first stage of the 2SLS model (Weak IV tests)

Variable	Simpson index
Internet use	0.083 (0.026)***
Anderson canon. corr. *LM*-statistic [P-val]	543.877 [0.00]
Cragg-Donald Wald *F*-statistic (10 % maximal IV size)	591.182 (16.38)

Note: Robust standard errors are presented in parentheses.

***< 0.01.
